# Randomised, split‐face study of a dermocosmetic cream containing *Sphingobioma xenophaga* extract and Neurosensine^®^ in subjects with rosacea associated with erythema and sensitive skin

**DOI:** 10.1111/srt.13735

**Published:** 2024-06-20

**Authors:** Enzo Berardesca, Claudia Cartigliani, Margot Nioré, Adriana Bonfigli, Ludivine Canchy, Delphine Kerob, Jerry Tan

**Affiliations:** ^1^ Phillip Frost Department of Dermatology University of Miami Miami USA; ^2^ Clinical Testing LabAnalysis Milano Italy; ^3^ Scientific Direction La Roche‐Posay Laboratoire Dermatologique Levallois‐Perret France; ^4^ Department of Dermatology Saint Louis Hospital Paris France; ^5^ Department of Medicine and Windsor Clinical Research Inc. Western University Windsor ON Canada

**Keywords:** dermocosmetic, erythema, external factors, rosacea, sensitive skin, skin barrier

## Abstract

**Introduction:**

Rosacea is a chronic inflammatory skin condition associated with erythema, inflammation and skin sensitivity.

**Objectives:**

To assess the benefit of a dermocosmetic cream (DC cream) containing *Sphingobioma xenophaga* extract and soothing agent in adult females with rosacea‐associated erythema and sensitive skin.

**Materials and Methods:**

During phase 1, DC was applied twice daily on the randomized half‐face and compared to usual‐skincare (USC) for 28 days. During phase 2, DC was applied on the full face twice daily for 56 days. Clinical, instrumental and skin sensitivity assessments were performed at all visits; demodex density (standardized skin surface biopsy (SSSB) method) was performed at baseline and D28, quality of life (QoL) was assessed using the stigmatization questionnaire (SQ), Rosacea Quality of Life index (ROSAQoL) and Dermatology Life Quality Index (DLQI) at baseline and D84.

**Results:**

At D28, a significant benefit of DC over USC was observed for erythema, tightness, burning and stinging (all *p* ≤ 0.05), erythema measured by chromameter (*p* < 0.01), corneometry and transepidermal water loss (*p* < 0.0001 and *p* < 0.05, respectively), skin sensitivity (*p* < 0.001) and significant reduction of mean demodex density (*p* < 0.05) on the DC side. At D84, DC significantly (all *p* < 0.05) improved clinical signs and symptoms on both sides of the face compared to baseline; SQ, ROSAQoL and DLQI scores improved by 40.4%, 25.0% and 55.7%, respectively compared to baseline. Tolerance was excellent.

**Conclusion:**

DC significantly improved erythema, skin sensitivity, demodex count, QoL and feeling of stigmatization of subjects with rosacea and is very well tolerated.

## 1 INTRODUCTION

Rosacea is a chronic inflammatory skin condition. It is commonly associated with an altered skin barrier, a dysbalanced microbiome, inflammation and vasodilation.^1^ Persistent erythema and telangiectasia are frequent primary features, and can be associated with flushing and papules, as well as sensitive skin symptoms.^2^, ^3^ The course of rosacea is fluctuant, with periods of flares and remission.^4^

In subjects with rosacea, the skin barrier is damaged and more sensitive towards external aggressions such as heat, cold and pollution.^7^, ^8^ These factors may lead to flares of rosacea and further skin discomfort.^9^, ^10^, ^11^ Symptoms of sensitive skin are frequently reported in rosacea and include skin burning, itching, stinging and tingling. Moreover, patients often feel uncomfortable and stigmatized, adding a psychosocial burden to the visible features of rosacea.^2^, ^3^ Several of these symptoms have been described as the secondary symptoms of rosacea.^1^

Rosacea is associated with a microbiome dysbiosis with increase in Demodex mites and expression of transient receptor potential cation channel subfamily V member 1 TRPV1, tropomyosin receptor kinase A (TrkA) and nerve growth factor (NGF), triggering cutaneous neurogenic inflammation.^5^ Rosacea associated with erythema and flushing and burning sensation refractory to traditional treatment may be designated as neurogenic rosacea.^6^

A specific dermocosmetic cream (DC cream, Toleriane Rosaliac^®^ AR concentrate cream, La Roche‐Posay Laboratoire Dermatologique, France) containing acetyl dipeptide‐1 cetyl ester (Neurosensine^®^), a soothing compound that acts on skin sensitivity by decreasing erythema and irritation as well as reducing signs related to neurogenic inflammation, and shea butter to moisturise the skin barrier, has been developed for rosacea patients.^12^ It also contains an extract of a flagellated bacterial strain of *Sphingomonas xenophaga* isolated from the endogenous flora component of La Roche‐Posay thermal spring water. It improves skin barrier function, decreases inflammation and inhibits, with a dose‐response effect, the activity of pre‐Kallikrein which plays an important role in inflammation.^13^ The increase in quantity and the magnitude of biological activity of Kallikrein 5 leads to the increased production of cathelicidins such as LL‐37. LL‐37 is an antimicrobial peptide that increases innate cutaneous inflammation, vasodilation and vascular proliferation, which are underlying pathogenic features of rosacea.^14^

This study assessed the benefit and local tolerance of DC cream in patients with rosacea associated with erythema and sensitive skin.

## 2 MATERIALS AND METHODS

This, randomized, controlled, double phase (phase 1: split‐face, phase 2: full face) study was conducted between January and May 2023 at one single site in Italy. The study adhered to the principles of Good Clinical Practices and the declaration of Helsinki. According to local regulatory guidelines, this type of study testing marketed cosmetics did not require approval from local ethics committees. However, the local ethics committee was informed about this study and subjects provided written informed consent prior to participation.

Female subjects > 18 years of age with a phototypes I–IV, almost clear (very few small papules/pustules, very mild erythema) to mild (few small or large papules/pustules, moderate erythema) rosacea (IGA 1–2), and sensitive skin (positive skin stinging test with 15% lactic acid) were recruited.^15^

During phase 1 and according to a randomisation schema, subjects applied DC cream on one half‐face and their commercially purchased usual skin care on the other half‐face (USC) twice daily, in the morning and evening, for 28 days. This comparative and randomised period was followed by a 56‐day period during which all subjects applied DC cream twice daily on the full face, up to visit D84.

Clinical evaluations at baseline, D15, D28 and D84 included erythema, skin tightness, burning sensation, stinging and pain, according to the VAS scale (0 = none to 10 = very much), rosacea severity (0 = none to 4 = severe; modified IGA Scale), stinging test and local tolerability.

Instrumental evaluations at all visits included chromametry (Chromameter CR400^®^, Konica Minolta, Japan) assessing erythema, corneometry (Corneometer CM825^®^, Courage & Khazaka, Germany) evaluating skin hydration, and transepidermal water loss assessments (TEWL; Tewameter TM 300^®^ MDD 4, Courage & Khazaka Germany).

Demodex density was assessed at Baseline and Day 28 on both sides of the face using the standardized skin surface biopsy (SSSB) method; microscopy pictures were taken using Dinolite^®^ AM, Dunwell Tech, USA with DinoCapture^®^ 2.0 software.^16^

Quality of life (QoL) was rated using the stigmatization questionnaire (SQ), ROSAQoL and DLQI at baseline and D84.^17^, ^18^, ^19^

Global tolerance was assessed at all post‐baseline visits on a scale from 0 = none to 3 = excellent tolerability.

Digital image capture was performed using ColorFace^®^ (Newtone Technologies, France) and Skincam^®^ (Newtone Technologies, France).

Mean values, standard deviations and variations were calculated. The ANOVA and the Bonferroni Tests were used to compare instrumental data at baseline, D15, D28 and D84. The t‐test was used to compare variations at D15‐baseline, D28‐baseline and D84‐baseline in the DC cream‐treated areas and in those having received USC. The Friedman ANOVA and Kendall's Concordance Coefficient Scores were used to compare clinical evaluations and the stinging test at baseline, D15, D28 and D84. The Wilcoxon test for non‐parametric and dependent data was used to compare variations at D15‐baseline, D28‐baseline, D84‐baseline and the Demodex density on both half‐face sides, as well as for the scores of stigmatization, DLQI and RosaQoL questionnaire at baseline and D84.

## 3 RESULTS

Twenty‐two women aged between 38 and 71 years, phototype II (11 subjects) or III (11 subjects) and an IGA grade of 1 (9 subjects) or 2 (13 subjects) were included in this study.

**FIGURE 1:**
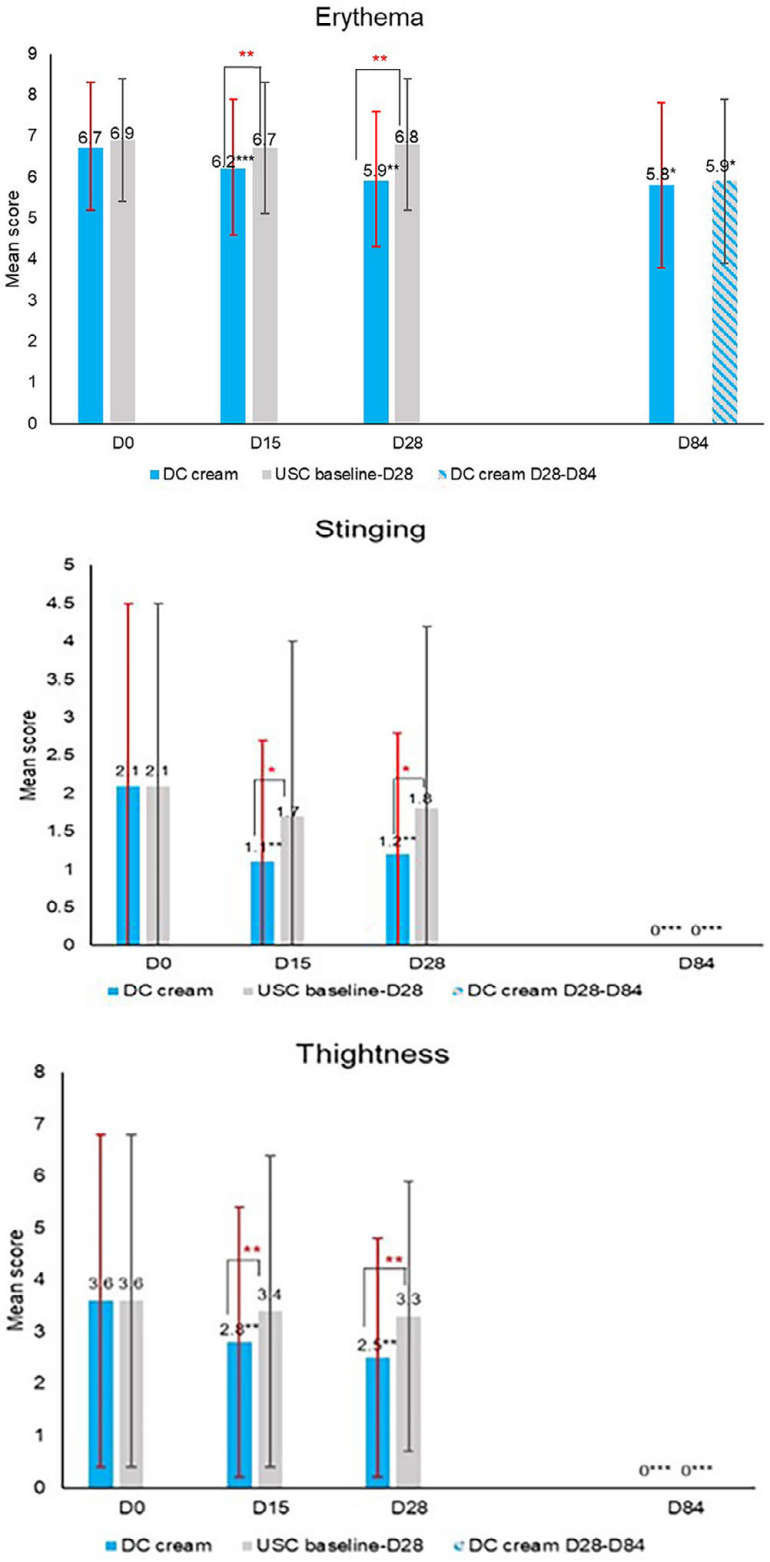
Clinical evaluation of signs and symptoms: mean scores for erythema, tightness, and stinging. *p < 0.05; **p < 0.01; ***p < 0.001.

**FIGURE 2:**
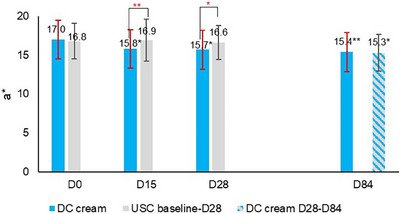
Instrumental assessment of erythema (mean values for a* parameter). *p < 0.01; **p < 0.001.

The clinical evaluation of erythema (Figure 1) showed that DC cream (D15: −7.5%, D28: −11.9%) compared to USC (D15: −2.9%, D28: −1.4%) significantly reduced erythema after 15 days (*p* < 0.001) and 28 days (*p* < 0.01) of use. At D84, erythema had significantly (*p* < 0.05) decreased on both sides of the face, with no between‐side difference. Chromameter assessments (Figure 2) showed a statistically significantly higher decrease with DC (D15: −7.0%, D28: −7.6%) than with the USC (D15: 0.5%, D28: −1.3%) at both time points (D15: *p* < 0.001; D28: *p* < 0.01), confirming clinical observations for erythema. Again, at the end of the study, erythema had significantly improved on both face sides (DC cream: *p* < 0.001, USC: *p* < 0.01) with no between‐side difference.

Skin tightness significantly (*p* < 0.01) decreased with DC cream by 22.2% and 30.6%, compared to 5.6% and 8.3% with the USC at D15 and D28, respectively. Similar findings were noted for burning (DC: −72.7% at D15 and D28, both *p* < 0.05 vs. USC: D15: −11.1%, D28: −22.2%) and stinging (DC: −47.6% at D15 and −42.9% at D28, both *p* < 0.01 vs. USC: D15:−19.0% and D28: −14.3%). Reductions were all significantly (*p* < 0.05) higher with DC cream compared to USC at both D15 and D28 time points, for which no significant difference from baseline was observed (Figure 1). At D84, all clinical symptoms had significantly improved on both sides compared to baseline (all *p* < 0.05) with no event of skin tightness, burning or stinging on both half‐faces.

Corneometer measurements showed a statistically significant (*p* < 0.0001) improvement of skin hydration with DC cream at D15 (+53.0% vs. +11.2% with USC), D28 (+59.2% vs. +12.2% with USC), continuing with DC cream until D84 (+65.7% vs. +67.3% with previous USC use). Improvements were significantly (*p* < 0.0001) higher with DC than with USC, at both D15 and D28, and similar at D84.

**FIGURE 3:**
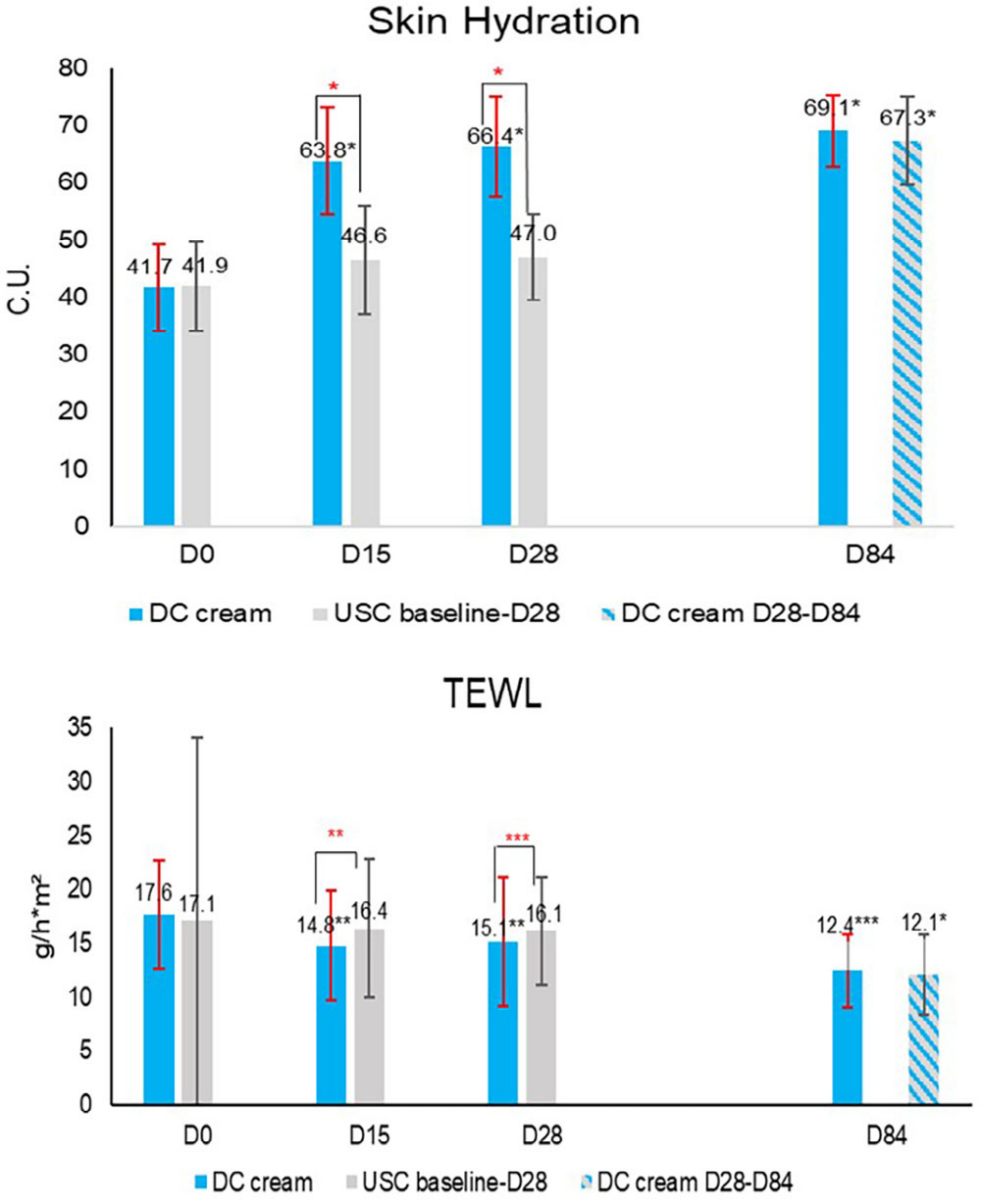
Instrumental assessment: mean units for skin hydration and TEWL. *p < 0.0001; **p < 0.001; ***p < 0.05.

TEWL (Figure 3) significantly decreased from baseline with DC cream at D15 (−16.2%; *p* < 0.01 vs. −4.2% with USC) and D28 (−14.5%; *p* < 0.05 vs. −5.6%), and continued to decrease with DC cream until D84. The decrease was significantly (*p* < 0.05) higher with DC cream than with USC at both D15 and D28. At D84, TEWL had further decreased on both face sides (*p* < 0.0001), with no between‐side difference.

**FIGURE 4:**
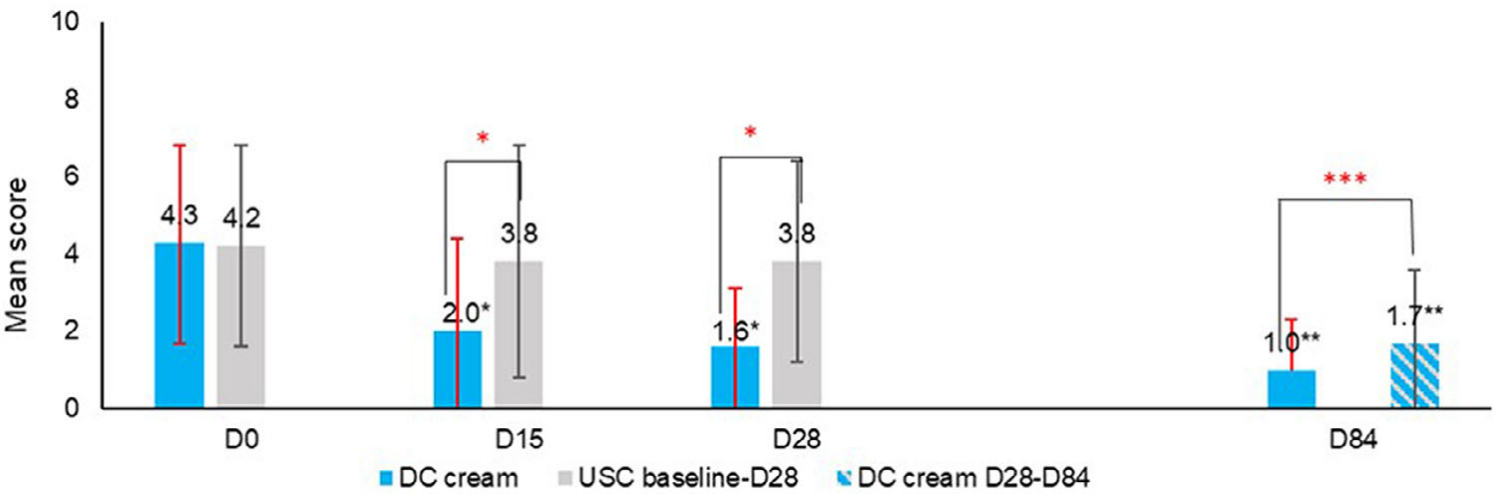
Skin sensitivity: mean scores. *p < 0.001; **p < 0.0001; ***p < 0.05.

Skin sensitivity (Figure 4) significantly decreased with DC cream at D15 (−53.5%; *p* < 0.0001 vs. −9.5% with USC) and at D28 (−62.8%; *p* < 0.001 vs. −9.5% with USC) from baseline. DC cream did significantly (*p* < 0.001) better than USC as early as D15. At D84, DC cream provided additional relief over the entire face (*p* < 0.0001).

**FIGURE 5:**
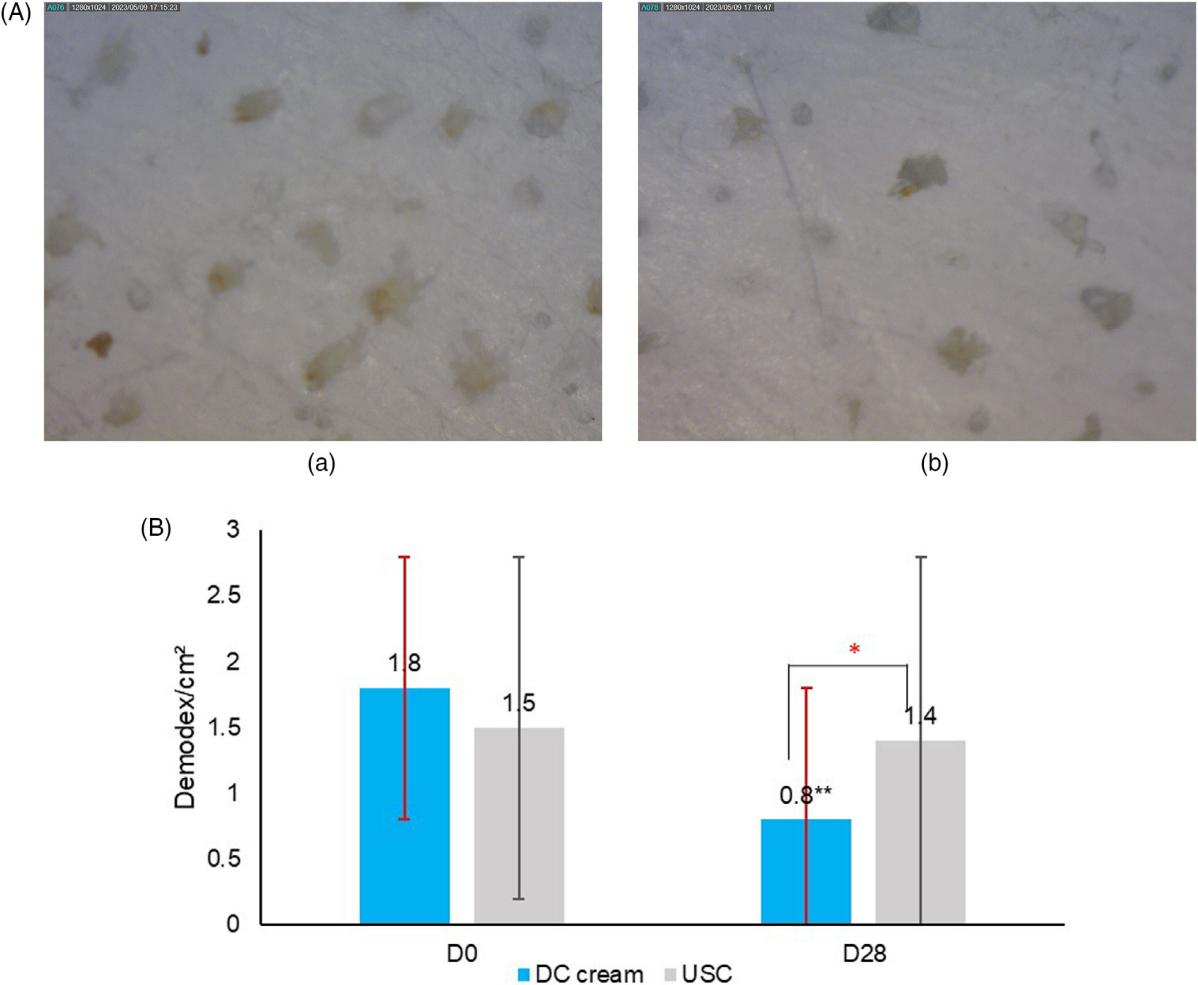
(A) Demodex density in microscopy at D28 (a) USC and (b) DC cream. (B) Improvement of mean Demodex density at D28 compared to D0. *p < 0.05; **p < 0.01.

A significant (*p* < 0.05) reduction of the mean Demodex density was observed on the DC cream side (−56.0%), compared to the USC side (−7%) at D28. Figure 5a provides microscopy pictures of the Demodex density and Figure 5b shows the evolution of the Demodex density at D0 and D28.

Results from the stigmatization questionnaire showed that the total score significantly (*p* < 0.001) decreased from 10.4 points at D0 to 6.2 points at D84 (−40.4% compared to baseline). So did the ROSAQoL (baseline: 2.8 points vs. D84: 2.1 points (−25.0%), *p* < 0.0001) and the DLQI (from 7.0 points to 3.1 points (−55.7%), *p* < 0.001).

Tolerance to DC cream was excellent in all subjects.

**FIGURE 6:**
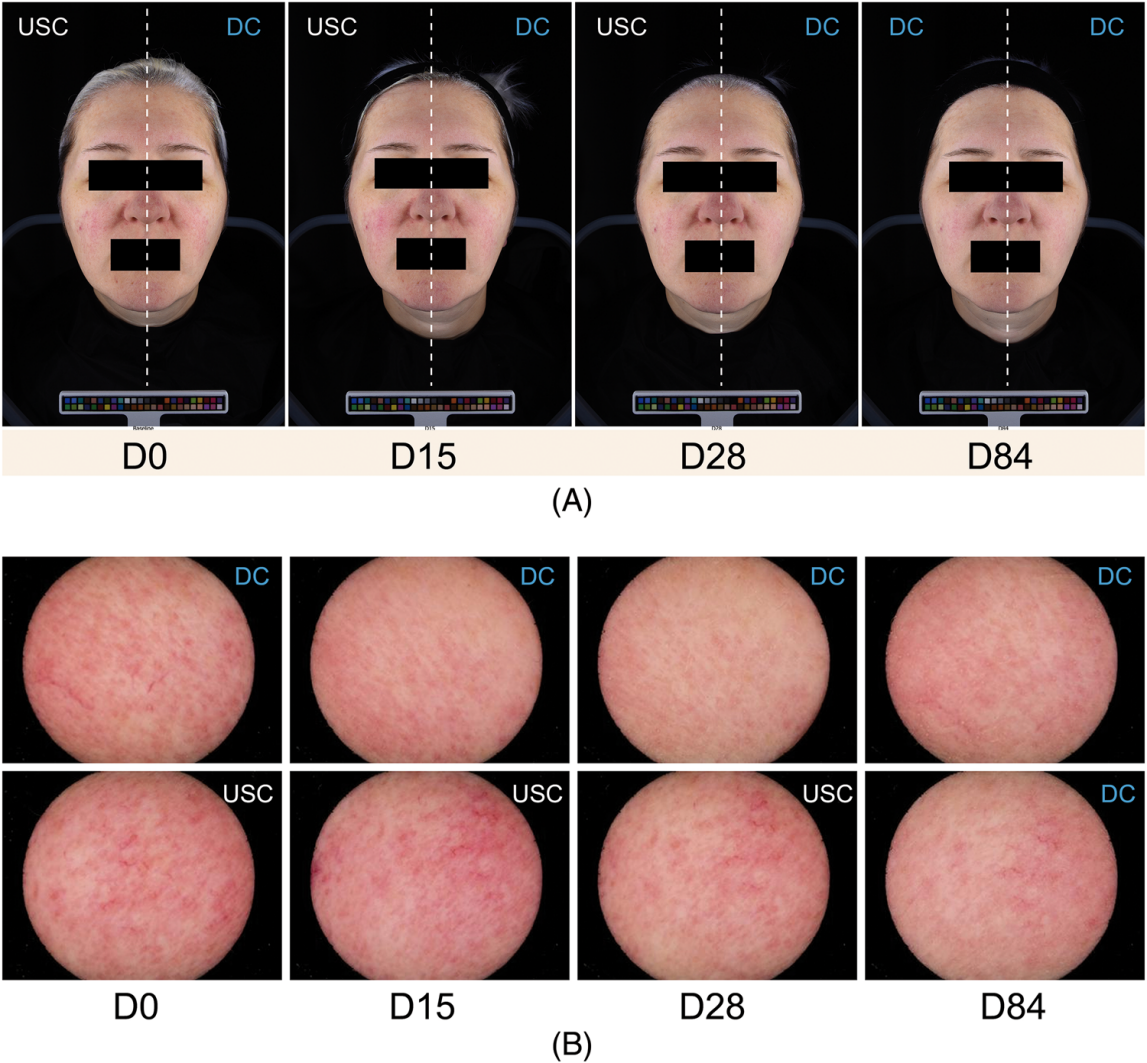
Images of rosacea using ColorFace^®^ and Skincam^®^; (A) ColorFace^®.^ (B) Skincam^®^. USC: usual skin care, DC: tested dermocosmetic cream

Figure 6 provides comparative ColorFace^®^ and Skincam^®^ images at D0, D15, D28 and D84.

## 4 DISCUSSION

Results from this study conducted in women with almost clear or mild rosacea associated with erythema, and sensitive skin showed that the tested dermocosmetic cream compared to the patients’ usual skin care significantly (all *p* < 0.05) improved erythema, rosacea signs and symptoms as well as skin sensitivity as early as after 15 days of daily use. Moreover, when switching after 28 days from USC to the DC cream, clinical and instrumental assessments confirmed that the DC cream was able to rapidly improve the condition of the half‐face previously having received the USC while continuing in parallel to improve the half‐face previously treated with the DC cream.

In rosacea, the natural skin barrier is damaged and neurogenic inflammation is activated.^20^ Dermocosmetics combined with a topical treatment or applied as a maintenance regimen are useful for subjects with rosacea, as they help to improve clinical signs and symptoms.^21^

The present study did not only show that the tested DC cream improved clinical signs and symptoms of rosacea compared to routine skin care, but also significantly (*p* < 0.05) improved physical skin barrier parameters, objective redness measured by chromametry, and reduced the density of Demodex after 28 days of daily use, thus helping to restore skin homeostasis. These improvements contributed to a significantly improved QoL and perception of social stigmatization, as shown after 84 days, thus confirming the added value of the tested DC cream compared to routine skin care in the management of very mild to mild rosacea.

The small sample size may be considered a limitation. However, due to the intra‐individual study design, the sample size of 42 hemi‐faces may be considered as sufficient to assess the benefits of the DC cream in rosacea compared to the subjects’ usual skin care regimen.

In conclusion, in the present study in subjects with rosacea associated with erythema and skin sensitivity, DC cream containing *S. xenophaga* extract and Neurosensine^®^ significantly improved signs and symptoms, regulated skin hydration and reduced the Demodex density compared to standard skin care. Moreover, the DC cream improved the subjects’ QoL and perception of social stigmatization and was very well tolerated.

## CONFLICT OF INTEREST STATEMENT

Margot Nioré, Ludivine Canchy and Delphine Kerob are employees of La Roche‐Posay Laboratoire Dermatologique, Jerry Tan receives consultancy honoraria from La Roche‐Posay Laboratoire Dermatologique. The other authors have no conflict of interest to disclose.

## ETHICS STATEMENT

This single centre, randomized study was conducted between January 2023 and May 2023 and adhered to the principles of Good Clinical Practices and the declaration of Helsinki. According to Italian regulatory guidelines, this type of trial testing marketed cosmetics does not require approval from local ethics committees. Even so, the local ethics committee of Milan/Italy was informed about this study and subjects provided written informed consent prior to participation including for the use of images.

## Data Availability

Enzo Berardesca, the corresponding author, will share the study protocol and all data collected and statistically analysed and in relationship with this study, except de‐identified participant data, upon reasonable request for one year after publication of this manuscript.

## References

[1] Thiboutot D , Anderson R , Cook‐Bolden F , et al. Standard management options for rosacea: the 2019 update by the national rosacea society expert committee. J Am Acad Dermatol. 2020;82(6):1501‐1510. 32035944 10.1016/j.jaad.2020.01.077

[2] Cardwell LA , Nyckowski T , Uwakwe LN , Feldman SR. Coping mechanisms and resources for patients suffering from rosacea. Dermatol Clin. 2018;36(2):171‐174. 29499801 10.1016/j.det.2017.11.013

[3] Halioua B , Cribier B , Frey M , Tan J . Feelings of stigmatization in patients with rosacea. J Eur Acad Dermatol Venereol. 2017;31(1):163‐168. 27323701 10.1111/jdv.13748

[4] Powell FC. The histopathology of rosacea: ‘where's the beef?’. Dermatology. 2004; 209:173‐174. 15459527 10.1159/000079884

[5] Lee SG , Kim J , Lee YI , Choi YS , Ham S , Lee JH . Cutaneous neurogenic inflammation mediated by TRPV1‐NGF‐TRKA pathway activation in rosacea is exacerbated by the presence of Demodex mites. J Eur Acad Dermatol Venereol. 2023;37(12):2589‐2600. 37606610 10.1111/jdv.19449

[6] Li M , Tao M , Zhang Y , Pan R , Gu D , Xu Y . Neurogenic rosacea could be a small fiber neuropathy. Front Pain Res (Lausanne). 2023;4:1122134. 36890854 10.3389/fpain.2023.1122134 PMC9986523

[7] Holmes AD , Steinhoff M . Integrative concepts of rosacea pathophysiology, clinical presentation and new therapeutics. Exp Dermatol. 2017;26(8):659‐667. 27376863 10.1111/exd.13143

[8] Hari A , Flach TL , Shi Y , Mydlarski PR . Toll‐like receptors: role in dermatological disease. Mediators Inflamm. 2010;2010:1. 10.1155/2010/437246 PMC2933899 20847936

[9] Damiani G , Gironi LC , Grada A , et al. COVID‐19 related masks increase severity of both acne (maskne) and rosacea (mask rosacea): Multi‐center, real‐life, telemedical, and observational prospective study. Dermatol Ther. 2021;34(2):e14848. 33533563 10.1111/dth.14848 PMC7995182

[10] Chiriac AE , Wollina U , Azoicai D . Flare‐up of Rosacea due to face mask in healthcare workers during COVID‐19. Maedica (Bucur). 2020;15(3):416‐417. 33312262 10.26574/maedica.2020.15.3.416 PMC7726500

[11] Gueniche A , Nielsen M . Introduction to probiotic fractions and Vichy volcanic mineralizing water: two key ingredients for stressed skin. J Eur Acad Dermatol Venereol. 2022;36 Suppl 2:3‐4. 10.1111/jdv.17783 34979588

[12] Boo YC . Mechanistic basis and clinical evidence for the applications of nicotinamide (niacinamide) to control skin aging and pigmentation. Antioxidants. 2021;10(8):1315. 34439563 10.3390/antiox10081315 PMC8389214

[13] Di Paolo CT , Diamandis EP . Prassas I. The role of kallikreins in inflammatory skin disorders and their potential as therapeutic targets. Crit Rev Clin Lab Sci. 2021;58(1):1‐16. 32568598 10.1080/10408363.2020.1775171

[14] Two AM , Del Rosso JQ . Kallikrein 5‐mediated inflammation in rosacea: clinically relevant correlations with acute and chronic manifestations in rosacea and how individual treatments may provide therapeutic benefit. J Clin Aesthet Dermatol. 2014;7(1):20‐25. PMC3930536 24563692

[15] Berardesca E , Farage M , Maibach H . Sensitive skin: an overview. Int J Cosm Sci. 2013;35(1):2‐8. 10.1111/j.1468-2494.2012.00754.x 22928591

[16] Yun CH , Yun JH , Baek JO , Roh JY , Lee JR . Demodex mite density determinations by standardized skin surface biopsy and direct microscopic examination and their relations with clinical types and distribution patterns. Ann Dermatol. 2017;29(2):137‐142. 28392639 10.5021/ad.2017.29.2.137 PMC5383737

[17] Tannus FC , Picosse FR , Soares JM , Bagatin E . Rosacea‐specific quality of life questionnaire: translation, cultural adaptation and validation for Brazilian Portuguese. An Bras Dermatol. 2018;93(6):836‐842. 30484528 10.1590/abd1806-4841.20187192 PMC6256224

[18] Van Beugen S , Schut C , Kupfer J , et al. Perceived stigmatization among dermatological outpatients compared with controls: an observational multicentre study in 17 European countries. Acta Derm Venereol. 2023;103:adv6485. 37345973 10.2340/actadv.v103.6485 PMC10296546

[19] Finlay AY , Khan GK . Dermatology life quality index (DLQI) – a simple practical measure for routine clinical use. Clin Exp Dermatol. 1994;19(3):210‐216. 8033378 10.1111/j.1365-2230.1994.tb01167.x

[20] Ivanic MG , Oulee A , Norden A , Javadi SS , Gold MH , Wu JJ . Neurogenic rosacea treatment: a literature review. J Drugs Dermatol. 2023;22(6):566‐575. 37276164 10.36849/JDD.7181

[21] Baldwin H , Alexis AF , Andriessen A , et al. Evidence of barrier deficiency in rosacea and the importance of integrating OTC skincare products into treatment regimens. J Drugs Dermatol. 2021;20(4):384‐392. 33852244 10.36849/JDD.2021.5861

